# 

**Published:** 1896-12

**Authors:** 


					﻿Vol. XVI.
DECEMBER, 1896.
No, 12.*
THE
HOMEOPATHIC pHY^lClAJl,
A Monthly Journal of Homoeopathic Materia Medica.
and Clinical Medicine.
“ is a freeman whom truth makes free, and all are slaves beside."
"Seek the Truth: come whence it may, cost what it will."
EDITED AND PUBLISHED BT
WALTER M. JAMES, M. D.
PHILADELPHIA :
nsro. 1231 LOCUST STREET.
London:
ALFRED HEATH & CO., 8ole Agents for Great Britain,
114 Ebury Street, Eaton Square.
Yearly Subscription, S2.6O, invariably in advance. Single numbers, »3 etc.
Entered at the Philadelphia Poet-Office at Second-Olaae Mail Matter.
				

